# Outdoor Poor Relief in Buffalo

**Published:** 1899-04

**Authors:** 


### OUTDOOR POOR RELIEF IN BUFFALO.

THE Charity Organisation Society of Buffalo has always stood for honesty, economy and a thorough and rigid administration of the various departments under its control. It was therefore not surprising when it undertook to ferret out some of the cases on the poor department rolls, a short time ago, that a startling condition of affairs was unearthed. The answer made by the overseer of the poor to these charges does not alter the state of affairs ; neither does it throw any doubt or detraction on the report as originally made by the Charity Organisation Society.

Briefly summarised the report shows that Buffalo gives more outdoor poor aid annually than the total given by New York, Philadelphia, Brooklyn, Baltimore, San Francisco, Washington, Louisville, Kansas City, St. Louis, New Orleans, Cincinnati, Jersey City, Pittsburg, Newark, Minneapolis and Cleveland combined—and with $10,000 to spare. The above cities comprise seventeen of the twenty-one largest cities in the United States which have a population of over 200,000. Buffalo not only gives more outdoor poor aid than the other sixteen combined, but taking the four omitted cities, Buffalo gives nearly as much poor relief as Chicago—a city five times its size—nearly twice as much as Boston, and more than Detroit and Milwaukee together.

The question may well be asked, what do we receive in return, or what do we hope to realise by such munificent charities? Appreciation, alleviation, the relief of pauperism and eventually its disappearance in whole or part, or does lavish outdoor relief spread the burden of caring for the poor upon all the taxpayers and so relieve private charity?

No—hardly any of these are realised, but as a superintendent of the poor, of fourteen years' experience has stated, public outdoor aid makes more paupers than even liquor, and that in cities where there is a liberal outdoor relief there is not less but more unrelieved poverty and more need of private charity than where there is none.

It is certain that outdoor relief is alluring, enticing, an active incentive to unthrift, or else a misappropriation of funds for personal excesses and vices in flush times and a haven for succor in times of paucity and the deserving as well as the undeserving poor turn toward the city treasury with a sense of right and justification—as a source of aid rightfully due them.

Charity has always covered a multitude of sins. Public charity, unless exercised with the most scrupulous care and supervision, is liable to foster, encourage and even protect a horde of sinners against law, morals and thrift; encouraging intemperance and improvidence, thereby producing results exactly the opposite which such charities had hoped to attain.

From a medical point of view, the charity organisation society is on the right track and we trust it will be highly successful in its crusade against the poor department. Nothing is so productive of disease as idleness. Idleness begets vice, filth, inaction of the various organs and consequently a lowered vital condition of the whole body

—hence it is that as a rule epidemics are to be found among the filthy and indolent. Many of the degenerate classes take their origin in the atmosphere of indolence and intemperance and the propagation of their kind and species adds still further burdens on the shoulders of those who are manfully trying to escape the stigma of public pauper.

The JOURNAL has on several occasions pointed out the wrong done by the expenditure of large sums of money not only to the taxpayers, to the thrifty poor, but to the city itself. It has pointed out the way at least in which some return might be expected from the money expended and thus help out at least the taxpayers to some extent. But this is not all, the plan proposed will do the poor just as much good as public charity, will sharpen his appetite, circulate his blood, tone up his muscle, diminish his tendency to disease and keep him out of mischief for ten hours a day at least, besides adding to the general beauty, welfare, health and comfort of the city and its inhabitants.

Let the appropriation to the poor department be turned over to the street department and let the former issue orders on the street cleaning department instead of on the grocer, coal dealer and others, and in that way we will lessen the number of public paupers, besides having some return for the money expended. The streets of Buffalo in winter are not one of the sources of pride and satisfaction to which one can point his finger, but are a menace and shame to one of the handsomest cities in the Union. What is needed is a Leonard Wood to direct and supervise an army composed of volunteers and drafted men who enlist annually at the poor department and who in the past have rendered as little public assistance as some of the drones he encountered in Santiago.

All those needy poor who are not able to work in the street cleaning department could be made to find some employment through the charity organisation society or through the private charities, which under the different denominations are doing such heroic work in this community. To those who will not work or are not inclined, let them be given the option of the almshouse—following the plan adopted by the town of Brookline, Mass., where the board of overseers spent $9,000 annually in out-door relief for 355 persons. After much opposition an almshouse was built, and when it was finished notice was given that thereafter outdoor relief would be given only in very exceptional cases. Within a year the number of persons relieved fell from 355 to 53, and yet there was never more than seven in the almshouse. There was no increased demand upon private charity, and there were no beggars. What happened? "Old men and women, whose children had been perfectly willing that they should have outdoor relief, suddenly found devoted sons, willing to support them rather than have them go to the poorhouse; men and women who had declared themselves too weak to work, recovered strength with marvelous rapidity; and the laborer, all of whose earnings had been barely enough to support him through the summer, now saved for the winter, and saved enough to keep him in tolerable comfort." What took place was what Dr. Chalmers, of Edinburgh, calls the development of natural resources.

The charity organisation society holds district committee meetings almost daily. At one of the meetings twenty-four applications for relief were considered. Fourteen of them were already receiving aid from the city, and in the case of ten of these fourteen families the committee thought the aid undeserved and harmful. At the next meeting of this same committee fifteen families were considered, eleven of which were receiving city aid. In the case of eight of these eleven the committee expressed its disapproval of the city aid.

It was found on investigation that the recipient of city alms in these cases were devotees of vice, drunkenness and profligacy and their eradication from the poor rolls was at once demanded. The charity organisation society deserves the thanks and good-will of the citizens of Buffalo and especially of the physicians, who are more or less intimately connected with the needy poor, both in and out of hospital and dispensary life. That this organisation may succeed in its warfare on public outdoor relief is one of the long-cherished hopes of the JOURNAL.

